# 

**Published:** 1908-08-29

**Authors:** 


### THE GRADUATES' MEDICAL SCHOOLS.

DIARY FOR WEEK AUGUST 31 to SEPTEMBER 5.

### THE POST-GRADUATE COLLEGE, West London Hospital, Hammersmith, W.

**At 12 noon.**
**August 31**, Dr. Low, **Pathological Demonstration.**
**At 5 p.m.**
**August 31**, Mr. Dunn, **Cases of Eye Diseases.**
**At 12.15 p.m.**
**September 2**, Dr. Pritchard, **Practical Medicine.**
**At 5 p.m.**
**September 2**, Dr. Robinson, **Gynæcology.**
**September 4**, Mr. Keetley, **Clinical Lecture.**

The rate of annual subscriptions to The Hospital, either direct from the Office or through agents, is :—

For the United Kingdom ... ... ... 15s. a year
To the Colonies and Abroad ... ... 17s. a year
inclusive of postage in each case.

### Forthcoming Publications.

A volume of occasional addresses by Dr. W. Osler, the Regius Professor of Medicine at Oxford, entitled "An Alabama Student and other Biographical Essays," will soon be published by the Oxford University Press. The greater portion of the book deals with aspects of the life of physicians in the United States, and Professor Osler is of opinion that in no age and in no land have the Hippocratic ideals been more fully realised than in some of the lives which he portrays. Chapters are devoted to Sir Thomas Browne, Harvey and his discovery, John Locke as a physician, "Keats the apothecary poet," and Oliver Wendell Holmes.

Messrs. John Wright and Company, of Bristol, will shortly publish "A Manual of Natural Therapy," by Thos. D. Luke, M.D., F.R.C.S. Ed., Lecturer at the University of Edinburgh. This work deals with medical baths, massage, electrotherapy, diet, the Weir-Mitchell treatment, etc. It will contain about 130 illustrations.

The same firm have also in the press "A Synopsis of Surgery," by Ernest W. Hey Groves, M.S., F.R.C.S., Assistant Surgeon to the Bristol General Hospital, price 7s. 6d. net; and the fifth edition of "Pye's Surgical Handicraft," edited and largely re-written by Wm. Henry Clayton-Greene, B.A., M.B., M.C. Cantab., F.R.C.S., Assistant Surgeon and Surgeon in Charge of Out-patents, St. Mary's Hospital, with nearly 300 illustrations, entirely re-drawn for this edition.

### The Health of Liverpool.

Dr. Hope, medical officer of health for the city of Liverpool, in his report for 1907, states that that year was characterised by the lowest death-rate ever recorded in Liverpool. The average rate for the decade ending 1870 was 32.5 per 1,000; during the next decade the average was 28.5, and in the next ten years 26.1. From 1891 to 1900 the average was 23.9, and there had been an almost steady decrease since, the average for the seven years being 20.4, and last year's rate being 18.3. During the same period of years the birth-rate reached its highest point in 1864 and 1865, when it was 40.8, and its lowest point last year, 31.7, as compared with 26.3 in England and Wales. Dr. Hope says that there was a gratifying decrease in infant mortality. The benefits of the general sanitary measures adopted in the city are nowhere more conspicuously shown than in the case of tuberculosis, the decline in this form of disease being one of the most encouraging features of urban sanitary administration.

